# Technology Use, Comfort, and Interest: A comparison between caregiver and older adult populations

**DOI:** 10.1093/geroni/igab046.2491

**Published:** 2021-12-17

**Authors:** Sarah Hubner, Akankshya Chataut, Marcia Shade, Ann Fruhling, Natalie Manley, Meaghan Fitzgerald Walls, Julie Blaskewicz Boron

**Affiliations:** 1 University of Nebraska Omaha, Omaha, Nebraska, United States; 2 University of Nebraska Medical Center, Omaha, Nebraska, United States; 3 University of Nebraska Omaha, University of Nebraska at Omaha, Nebraska, United States

## Abstract

Remaining community-dwelling is a goal for most aging adults; however, this may necessitate assistance from caregivers. To reduce burden and improve adult autonomy, recent technological advancements have provided various supports. These advancements may improve quality of life (QOL) while also enhancing psychological/physical well-being for adults and caregivers. To investigate relationships between technology, QOL, and caregiver burden, needs assessments with focus groups were utilized. Four older adult focus groups (N=20) and three caregiver focus groups (N=12) were convened. Older adult participants, aged 64-83 years (M=73.1,SD=5.3), were 50% female and generally white (90%). Caregiver participants, aged 31-78 years (M=61.9,SD=12.6), were majority female (83%) and generally white (92%). Because of the ongoing COVID-19 pandemic, focus groups were conducted via Zoom video-conferencing. Thematic analyses revealed major themes of privacy, transportation, and interest in streamlined technologies. Throughout groups, privacy was consistently described; participants were either 1) apathetic, noting absence of privacy or 2) hyper-vigilant about security, citing privacy as a major barrier to utilization. Transportation, specifically self-driving/enhanced vehicles, emerged as a focus for future technologies as a means to reduce care burden and improve personal autonomy/QOL. In general, participants noted that major barriers to technology use included complexity and cost; persons expressed interest in simpler/cheaper devices. This study indicates varied interest in technology while exposing barriers to use. Additionally, the methodology demonstrates the utility of technology (e.g., Zoom) in accessing vulnerable and/or isolated populations. Overall, understanding barriers to technology use and adoption informs upcoming developments and may improve accessibility and usefulness in future systems/devices.

